# Triplanar estimation of left atrial volume

**DOI:** 10.1186/1532-429X-13-S1-P327

**Published:** 2011-02-02

**Authors:** Pedram Kazemian, June Baron, Kelvin Chow, Mark Haykowsky, Richard Thompson, Ian Paterson

**Affiliations:** 1 University of Alberta, Edmonton, AB, Canada

## Introduction

Left atrial volume (LaV) is an important predictor of outcome for many cardiovascular conditions. The most commonly used method to estimate LaV is the area-length (AL) biplane formula based upon LA measures from 2 and 4 chamber (CH) views. Given the asymetrical shape of the LA, we hypothesized that a formula incorporating measures from all 3 conventional long-axis views is a more accurate method for estimating LaV.

## Purpose

The aim of this study was to assess the accuracy of AL biplane formula for LaV and to derive a new formula for estimating LaV based on measurements from the two, three and four CH views.

## Methods

Consecutive atrial fibrillation (AF) patients referred for CMR to assess pulmonary veins and left atrial size prior to catheter based treatment were included. During the study period, patients without AF and minimal structural heart disease on CMR were also examined. For each patient group, a 1.5T magnet was used to acquire 2, 3 and 4 CH cines in addition to an axial stack through the heart. Left atrial dimensions, width (W) and length (L), and areas (A) were measured on each of the three long axis views and the LaV was estimated using the AL biplane formula. The axial stack was used to calculate the true LaV by a method of disks (MoD) summation. To find the optimum formula for estimating LaV, a full quadratic model equation was used. The regressor variables included dimensions and area of the left atrium obtained from each of the three long axis views. All calculated LaVs were compared to the true LaV to assess for accuracy.

## Results

Left atrial measures were assessed on 97 patients, 49 with AF, 67 male with mean age of 46±15. The LaV estimated by the AL biplane formula (mean 87.8±31.7 ml) was significantly lower than that derived from the MoD (mean 117.7±36.1 ml, p<0.001). Using Bland-Altman method, the 95% limits of agreements (LOA) were -69.5 ml to 9.6 ml. Our derived triplanar formula, LaV = 0.628 X Lmax X Amax, correlated better with MoD LaV (R^2^=0.79), than the AL biplanar method (R^2^=0.69) and provided an unbiased estimate of the true mean LaV, 95% LOA of -33.8 ml to 30.4 ml.

## Conclusions

A triplanar formula that considers measures from all three conventional long-axis views significantly improves accuracy of LaV measurement over the AL formula.

